# **RETRACTION** : Mechanism of ARPP21 antagonistic intron miR‐128 on neurological function repair after stroke

**DOI:** 10.1002/acn3.52086

**Published:** 2024-05-20

## Abstract

**RETRACTION**: Chai, Z., Zheng, P., Zheng, J. (2021) Mechanism of ARPP21 antagonistic intron miR‐128 on neurological function repair after stroke*. Annals of Clinical and Translational Neurology,* 8: 1408–1421. https://doi.org/10.1002/acn3.51379

The above article, published online on 28 May 2021 in Wiley Online Library (wileyonlinelibrary.com), has been retracted by agreement between the journal Editor‐in‐Chief Ahmet Hoke and Wiley Periodicals LLC on behalf of the American Neurological Association. The retraction has been agreed following a report by a third party who found that images in Figure 6 of the above article had been published previously in the following articles by other authors: Zhao et al. 2019 (1) and Shao et al. (2). Additionally, images from Figure 4B in the above article had been previously published by other authors in Zhao et al. 2020 (3).

The authors did not respond to an inquiry by the publisher and requests to supply original data and images. Because the authors did not provide an explanation or original data for review, the data reported in this article is considered unreliable and the journal must issue a retraction. The authors did not respond to our notice of retraction.

References:

(1) ZhaoX‐H., , WangY‐B., , YangJ., , LiuH‐Q., , WangL‐L, . (2019) MicroRNA‐326 suppresses iNOS expression and promotes autophagy of dopaminergic neurons through the JNK signaling by targeting XBP1 in a mouse model of Parkinson's disease. J Cell Biochem *,* 120:14995–15006. 10.1002/jcb.28761.31135066

(2) ShaoC‐Z, ., XiaK‐P, . (2018) Sevoflurane anesthesia represses neurogenesis of hippocampus neural stem cells via regulating microRNA‐183‐mediated *NR4A2* in newborn rats. J Cell Physiol *,* 234: 3864–3873. 10.1002/jcp.27158.30191980

(3) ZhaoP., , WangY., , ZhangL., , ZhangJ., , LiuN., , WangH., (2020) Mechanism of long non‐coding RNA metastasis‐associated lung adenocarcinoma transcript 1 in lipid metabolism and inflammation in heart failure. Int J Mol Med *,* 47: 5. 10.3892/ijmm.2020.4838.PMC783495833448307

**RETRACTION**: Chai, Z., Zheng, P., Zheng, J. (2021) Mechanism of ARPP21 antagonistic intron miR‐128 on neurological function repair after stroke*. Annals of Clinical and Translational Neurology,* 8: 1408–1421. https://doi.org/10.1002/acn3.51379

The above article, published online on 28 May 2021 in Wiley Online Library (wileyonlinelibrary.com), has been retracted by agreement between the journal Editor‐in‐Chief Ahmet Hoke and Wiley Periodicals LLC on behalf of the American Neurological Association. The retraction has been agreed following a report by a third party who found that images in Figure 6 of the above article had been published previously in the following articles by other authors: Zhao et al. 2019 (1) and Shao et al. (2). Additionally, images from Figure 4B in the above article had been previously published by other authors in Zhao et al. 2020 (3).

The authors did not respond to an inquiry by the publisher and requests to supply original data and images. Because the authors did not provide an explanation or original data for review, the data reported in this article is considered unreliable and the journal must issue a retraction. The authors did not respond to our notice of retraction.

**References:**

(1) Zhao, X‐H. , Wang, Y‐B. , Yang, J. , Liu, H‐Q. , Wang, L‐L . (2019) MicroRNA‐326 suppresses iNOS expression and promotes autophagy of dopaminergic neurons through the JNK signaling by targeting XBP1 in a mouse model of Parkinson's disease. J Cell Biochem *,* 120:14995–15006. 10.1002/jcb.28761.31135066

(2) Shao, C‐Z ., Xia, K‐P . (2018) Sevoflurane anesthesia represses neurogenesis of hippocampus neural stem cells via regulating microRNA‐183‐mediated *NR4A2* in newborn rats. J Cell Physiol *,* 234: 3864–3873. 10.1002/jcp.27158.30191980

(3) Zhao, P. , Wang, Y. , Zhang, L. , Zhang, J. , Liu, N. , Wang, H. (2020) Mechanism of long non‐coding RNA metastasis‐associated lung adenocarcinoma transcript 1 in lipid metabolism and inflammation in heart failure. Int J Mol Med *,* 47: 5. 10.3892/ijmm.2020.4838.PMC783495833448307

